# Group psychological counseling to contrast academic burnout: a research protocol for a randomized controlled trial

**DOI:** 10.3389/fpsyg.2024.1400882

**Published:** 2024-07-31

**Authors:** Irene Messina, Tatiana Rossi, Flavia Bonaiuto, Gianluigi Granieri, Paola Cardinali, Irene Petruccelli, Roberto Maniglio, Claudio Loconsole, Pietro Spataro

**Affiliations:** Faculty of Social and Communication Sciences, Mercatorum University, Rome, Italy

**Keywords:** academic burnout, randomized controlled trial, psychological counseling, group counseling, online counseling

## Abstract

Academic burnout is a condition characterized by exhaustion, cynicism, a distant attitude toward studying, and diminished self-efficacy in academic activities. Preliminary scientific findings indicate that interventions designed to alleviate work burnout also hold promise for mitigating academic burnout, however clear evidence based on randomized controlled trials is still missing. This research protocol describes a randomized controlled trial aimed at evaluating the efficacy of an online group psychological intervention to contrast academic burnout. Participants with high levels of burnout will be assigned to a psychological counseling group or a waiting list control group. The research comprises several phases: (T0) Screening, Recruitment, and Randomization; (T1) Baseline assessment (pre-intervention); (T2) Outcome Assessment (post-intervention); and (T3) Follow-up Assessment (3 months post-intervention). The primary outcomes include burnout symptoms, general wellbeing, and academic achievement. Additionally, secondary variables such as effort-reward imbalances, satisfaction/frustration of basic psychological needs, intrapersonal and interpersonal emotion regulation, coping strategies, and social support will be examined. The psychological intervention strategies will encompass psychoeducation, self-awareness enhancement, cognitive restructuring, and promotion of social support. This research protocol is an initial step toward evidence-based psychological interventions to treat academic burnout.

## Introduction

Burnout is a syndrome characterized by emotional exhaustion, depersonalization, and reduced personal accomplishment, traditionally attributed to chronic responses to occupational and interpersonal stress conditions at work (Freudenberger, 1974; Maslach, 1976). More recently, the concept of burnout has been extended to the educational context, where “academic burnout” is described as a condition characterized by exhaustion from studying, cynicism, distant attitude toward studying, and reduced self-efficacy in relation to academic activities (Schaufeli et al., 2002; Zhang et al., 2007). It is associated with a lower feeling of efficacy in tackling the course of studies (Rahmati, 2015), lengthening or interruption of the academic career (Madigan and Curran, 2021), as well as a higher incidence of psychological disorders (Deeb et al., 2018), substance abuse (Kadhum et al., 2022), suicidal ideation and suicide (Dyrbye et al., 2008).

The efficacy of psychological interventions to contrast work burnout has been largely demonstrated in several meta-analyses focused on general employees' categories (Maricutoiu et al., 2016; Ahola et al., 2017; Perski et al., 2017) and on more at-risk categories, such as health workers (Rahmati, 2015; Panagioti et al., 2017) and teachers (Iancu et al., 2018). Effective individual-centered interventions include awareness enhancing strategies - typically, self-monitoring of stress symptoms and psychoeducation on adaptive/maladaptive strategies to cope against stress—and arousal reductions strategies (for example, relaxation techniques and mindfulness meditation) (Le Blanc and Schaufeli, 2008). In more complex interventions, also strategies aimed at changing maladaptive cognitions have been successfully used (Le Blanc and Schaufeli, 2008; Bresó et al., 2011). Namely, negative cognitions concerning self-efficacy have been shown to play a key role, with high level of self-efficacy acting as a protective factor against burnout (Shoji et al., 2016).

To our knowledge, there is limited research on the efficacy of psychological interventions to treat academic burnout. Early scientific evidence suggests that psychological interventions to decrease work burnout are also promising for academic burnout. For instance, reduction of academic burnout scores has been documented after psychological counseling sessions to enhance self-awareness (Luo X., 2012; Luo Z. Y., 2012; Liu, 2013; Zhang et al., 2014; Guo, 2016; Shen, 2020), contrast maladaptive cognitions or “cognitive restructuring” (Ni and Wu, 2009; Xiong and Fang, 2017; Apriliya and Lianawati, 2023) or decrease stress related arousal (Ye et al., 2017; Wan, 2020).

In combination with individual-centered approaches, organizational approaches focused on enhancing social support may also be effective. Indeed, social support has both indirect (buffering) and direct effects in the relationship between stressors (e.g., academic demands) and students' burnout (Kim et al., 2018; Kusuma et al., 2022). Furthermore, strategies of interventions based on the provision of supportive relationships in the academic context could be useful to decrease students' burnout (Ezenwaji et al., 2019; Xu, 2019). Beyond specific strategies to enhance social support, the provision of psychological interventions in a group format could represent a natural source of social support (Biolcati et al., 2017; Brusadelli et al., 2020). Together with advantages in terms of social support provision, psychological interventions at the group level are the most suitable approaches for organizational settings. Indeed, group counseling is widely used in academic burnout interventions (Tang et al., 2021). More recently, early documentation of the efficacy of online group psychological counseling to treat academic burnout has been also provided (Eseadi, 2022; Apriliya and Lianawati, 2023).

In summary, early studies support the hypothesis that psychological interventions are useful to contrast academic burnout. Among others, interventions focused on self-awareness, cognitive restructuring, and social support are promising. Interestingly, there is also preliminary evidence that combining two or more strategies in integrated approaches could add value in terms of effectiveness of such interventions (Tang et al., 2021). However, the number of existing studies is still exiguous and available studies present relevant methodological limitations, above all the lack of adequate of control conditions (Tang et al., 2021). Moreover, the studies described above come almost exclusively from Asia and the adaptation to different cultural context cannot be taken for granted. Thus, more research is required in the direction of validating psychological interventions to treat academic burnout.

In the present research protocol, we propose a randomized controlled study designed to test the efficacy of an online group psychological intervention protocol to contrast academic burnout. We opted for Transactional Analysis (TA) as the theoretical background of this intervention (Berne, 1961; Vos and van Rijn, 2021). Even if TA has received limited attention by psychotherapy research, it is widely taught and practiced internationally within recognized academic and professional institutions (Vos and van Rijn, 2022), and it has a large history of application in organizational settings in Europe and United Kingdom (for organizational TA manuals see: Davidson and Mountain, 2016; Cannavale and Castagna, 2018; van Poelje and de Graaf, 2021). The main advantage of TA-based interventions for burnout lies in its intrinsic combination of individual and organizational approaches (Thunnissen and Timmermans, 2023), due to the simultaneous focus on human personality (focus on intra-personal processes) and social behavior (focus on interpersonal processes (Berne, 1961). This double focus is evident in the traditional group format of TA in its origins (Berne, 1958). Finally, TA treatments were one of the first models of psychological intervention that integrated cognitive-behavioral approaches with psychoanalytic concepts, developing a substantive new theory, as a strategy for integration (Vos and van Rijn, 2023). Thus, it is a flexible model, easily adaptable to the integration of different strategies of intervention, as in the psychological counseling program tested in the present research protocol.

### Aims, hypotheses, and study design

The present study is a randomized clinical trial aimed at generating evidence in support of the effectiveness of an online group psychological counseling intervention in university students with high levels of academic burnout. To this aim, the study design entails the comparison between students that will be randomly assigned to two matched groups: (1) a psychological counseling group (PC, *N* = 65) or (2) a waiting list control group (WL, *N* = 65). We will test the effect of time (pre-PC vs. post-PC) in interaction with the effect of group (PC vs. WL) on the score obtained in several self-report outcome measures, including scores on burnout symptoms, general wellbeing, and academic achievement as primary outcomes. We hypothesize that PC, compared to WL, should provide evidence of the efficacy of psychological counseling to decrease burnout symptoms and improve general wellbeing and academic achievement.

Moreover, to acquire data on possible mediators or psychological change, we will also assess a large set of secondary outcome variables potentially related to academic burnout, including effort-reward imbalances, basic psychological needs of satisfaction/frustration, intrapersonal and interpersonal emotion regulation, coping strategies and social support. Such variables will be directly and/or indirectly addressed in the psychological counseling sessions (see Section “Intervention” and Table 1). We hypothesize that secondary outcomes variables may be affected by the psychological counseling intervention, mediating or moderating its effects on primary outcomes.

**Table 1:** Summary of psychological counseling intervention features.

**Session**	**Strategy**	**In-session activities**	**Treatment goals**	**TA techniques**
First	Psychoeducation	Information provision on stress, burnout, and coping Information provision about intervention action and goals	Self-awareness	Contract
	Agreement treatment goals	Set goals for time in treatment	Self-awareness	
	Promotion of social support	Introduction of participants and personal experiences sharing	Social support	Therapeutic alliance/group cohesion
Second	Psychoeducation	Information provision on psychological basic needs Identification of basic needs and their satisfaction or frustration in the academic context	Self-awareness	Strokes Therapeutic alliance/group cohesion
	Promotion of social support	Experiential exercise of need satisfaction in the group Identification of adaptive ways to ask for social support	Social support	
Third	Promote self-awareness of personal reactions to stressful events.	Identifications of personal reaction (thoughts, emotions, somatic sensations, behaviors) to specific stressful events in the academic context.	Self-awareness	Ego states identification
	Promote self-awareness unhelpful reactions and development of more helpful reactions to stressful events.	Identification of unhelpful reactions to specific stressful events in the academic context, and initial development of more helpful reactions.	Cognitive restructuring	Ego states decontamination
Fourth	Promote self-awareness of mental representations of self and others.	Identifications of thoughts about the self and the other in the academic context (for example professors, university technical staff, or other students) and the related emotions, somatic sensations, and behaviors.	Self-awareness	Existential positions
	Development of helpful mental representations of self and others.	Explore the options for alternative mental representations of self and others.	Cognitive restructuring	
Fifth	Promote self-awareness of automatic behaviors.	Identifications of learning styles. Explore the options for alternative learning styles.	Self-awareness	Drivers
Sixth	Program review	Identify positive outcomes for each individual and areas they still want to work on.	Self-awareness	Contract completion
		Identify positive relationships in the group and future plans.	Social Support	

The research protocol was registered on ClinicalTrial.gov on January 2024 (protocol 9-FIN/RIC).

## Sample recruitment process

### Study setting

The study will be conducted at Mercatorum University, a private Italian online university. Due to the online nature of Mercatorum University, the psychological counseling interventions and the related research activities will be carried out online (in videoconference), in the context of the psychological counseling service of the university and using Google Meet links provided in the University Web Platform.

### Participants and sample size

Assuming a medium effect size (Cohen's *d* = 0.50), we estimated with the G^*^Power3 software (Faul et al., 2007) that we need a sample size of 128 participants (i.e., two groups of 64 students) to achieve a power of 0.80 (α = 0.05). Thus, a total of 130 students will be recruited from bachelor's courses at Universitas Mercatorum. Participants are eligible for inclusion according with the following criteria: (a) age > 18 years; (b) clinical levels of academic burnout scores [one standard deviation above the mean MBI-SS total score reported in the validation study by Portoghese et al. (2018)]; (c) attendance of 1^st^ or 2^nd^ year of bachelor course at Universitas Mercatorum; and (d) sufficient knowledge of the Italian language. Additional exclusion criteria aimed at selecting a sufficiently homogeneous group of participants are: (a) previous university degree; (b) other psychiatric diagnoses; (c) other ongoing psychological or psychiatric treatments. Finally, we also excluded (d) attendance of 3^rd^ year of bachelor course, to avoid participants drop-outs due to the course conclusion.

## Instruments

### Screening instruments

#### Maslach burnout inventory-student survey

The MBI-SS (Schaufeli et al., 1996; Italian adaptation: Portoghese et al., 2018) is a 15-item self-report questionnaire composed of three subscales: Exhaustion (EX; item example “*I feel used up at the end of a day at university*”), Cynicism (CY; item example “*I doubt the significance of my studies*”), and Professional Efficacy (PE; item example “*During class I feel confident that I am effective in getting things done*”). All the items are scored by using a 7-point Likert scale ranging from 0 (=“*never*”) to 6 (=“*always*”). We will use the Italian version of MBI-SS from the study of Portoghese et al. (2018), in which reliability coefficients for each of the subscale scores were 0.86 for EX, 0.82 for CY and 0.77 for PE. The MBI-SS will be used to select students with high levels of academic burnout.

#### Personal information and eligibility form

This form was developed to collect information about the demographic characteristics of the students participating in the study. The form contains a total of 10 questions regarding basic personal information (sex, age, previous experience of psychological counseling/psychotherapy, current, or previous psychological/psychiatric diagnoses), educational information (attended bachelor course, previous academic experience, previous academic degree, and parents' education), and economical status (current economic situation and economic situation in childhood).

### Primary outcomes measures

#### Maslach burnout inventory-student survey

The MBI-SS will be also used as primary outcome measure.

#### Psychological general wellbeing index–short version

The PGWB is a self-report measure of intra-personal affective or emotional states, which captures a subjective perception of wellbeing referring to the last 4 weeks (original version: Dupuy, 1984; Italian short-form version: Grossi et al., 2006). It is composed by items assessing Anxiety, Depressed Mood, Positive Wellbeing, Self-Control, General Health, and Vitality (item example “*How much energy, pep, or vitality did you have or feel during the past month?*”). All the items are scored by using a 6-point Likert scale ranging from 0 (for example “*Very full of energy–lots of pep*”) to 6 (for example “*No energy or pep at all–I feel drained, sapped*”).

#### Academic achievement

The academic achievements of the students participating to the study will be measured based on the self-reported number of passed exams and the average exam grades. Moreover, self-reported satisfaction in academic achievement will be also assessed with a 4-point Likert scale item ranging from 1 (=“*not satisfied*”) to 4 (=“*completely satisfied*”).

### Mediational measures

#### Basic psychological need satisfaction and frustration scale

The BPNSFS is a self-report general measure of the satisfaction and frustration of the psychological needs in one's life (Italian adaptation: Costa et al., 2018; Cardella et al., 2020). It is composed of the following subscales: Autonomy Satisfaction (item example “*I have a feeling of choice and freedom in what I do at work”*), Competence Satisfaction (item example “*I feel confident that I can do things well at work*”), Relatedness Satisfaction (item example “*I feel closely connected to other people who are important to me at work.”*), Autonomy Frustration (item example “*My daily activities at work feel like a continuous line of duties*”), Competence Frustration (item example “*I seriously doubt whether I can do things well at work ”*), and Relatedness Frustration (item example “*I feel that the relations I have at work are only superficial”*). All 24 items are scored on a 5-point Likert-scale ranging from 1 (=“*completely disagree*”) to 5 (=“*completely agree*”).

#### Effort-reward imbalance student questionnaire

The ERI-SQ is a self-report measure to identify effort-reward imbalance in university students (Wege et al., 2017; Italian adaptation: Portoghese et al., 2019). It is composed of the following subscales: Effort (item example “*I have constant time pressure due to a heavy study load”*), Reward (item example “*I receive the respect I deserve from my supervisors (teachers”*), and Over-Commitment (item example “*Student work rarely lets me go; it is still on my mind when I go to bed”*). All the items are scored by using a 4-point Likert scale ranging from 1 (=“*strongly disagree*”) to 4 (=“*strongly agree*”).

#### Cognitive emotion regulation questionnaire-short version

The CERQ-18 is a self-report measure of cognitive emotion regulation strategies individuals may use after experiencing negative life events (Garnefski and Kraaij, 2006; Italian adaptation: Cerolini et al., 2022). It evaluates the habitual use of Self-Blame (item example “*I feel that I am the one to blame for it”*), Other-Blame (item example “*I feel that others are to blame for it”*), Rumination (item example “*I often think about how I feel about what I have experienced”*), Catastrophizing (item example “*I often think that what I have experienced is much worse than what others have experienced”*), Putting Into Perspective (item example: “*think that other people go through much worse experiences”*), Positive Refocusing (item example “*I think of something nice instead of what has happened”*), Positive Reappraisal (item example “*I think I can learn something from the situation”*), Acceptance (item example “*I think that I have to accept that this has happened”*), and Refocus on Planning (item example “*I think about how I can best cope with the situation”*). All 18 items are scored on a 5-point Likert-scale ranging 1 (=“*almost never*”) to 5 (=“*almost always*”).

#### Difficulties in interpersonal emotion regulation

The DIRE is a self-report measure of maladaptive interpersonal emotion regulation strategies that may relate to psychopathology (Dixon-Gordon et al., 2018; Italian adaptation: Messina et al., 2022). It is composed of the following subscales: Venting (item example “*Raise your voice or criticize your friends to express how you feel”)*, Reassurance-Seeking [item example “*Keep contacting (texting, calling, etc.) friends and loved ones”*], Avoidance (item example “*Avoid feeling or showing your distress”*), and Acceptance (item example “*Simply notice your feelings”*). All 21 items are scored on a 5-point Likert-scale ranging from 1 (=“*very unlikely*”) to 5 (=“*very likely*”).

#### Coping orientation to problems experienced (Brief-Cope)

The Brief-Cope is a self-report measure of different coping reactions (Carver et al., 1989; Italian adaptation Bongelli et al., 2022). It includes the following subscales: Problem-Focused Coping (item example “*I've been trying to come up with a strategy about what to do”*), Emotion-Focused Coping (item example “*I've been getting emotional support from others”*), and Avoidant Coping (item example “*I've been using alcohol or other drugs to make myself feel better”*). All 28 items are scored on a 5-point Likert-scale ranging from 1 (=“*I haven't been doing this at all”*) to 4 (=“*I've been doing this a lot*”).

#### The multidimensional scale of perceived social support

The MSPSS is a self-report measure of perceived emotional support (Zimet et al., 1988; Italian Adaptation Cipolletta et al., 2022). Perceived social emotional support is evaluated over three dimensions: Family (item example “*My family really tries to help me”*), Friends (item example “*I can count on my friends when things go wrong”*), and Significant Others (item example “*I have a special person who is a real source of comfort to me”*), with each subscale containing four items. All the items are scored by using a 7-point Likert scale ranging from 1 (=“*strongly disagree*”) to 7 (=“*strongly agree*”).

### Other outcome variables

Together with self-rated improvements, additional indicators of outcome will be considered. First, acceptance rate will be calculated by taking the total number of participants who accepted, agreed, and consented to participate in this study and dividing it by the number of participants who were invited to participate. Second, adherence rate will be calculated as the total number of participants who completed the intervention according to the study protocol divided by the number of participants who started the intervention. Third, dropout rate will be calculated as number of participants who withdrew from or did not continue the intervention divided by the number of participants who consented to participate on the study.

## Procedures

**Figure 1:**
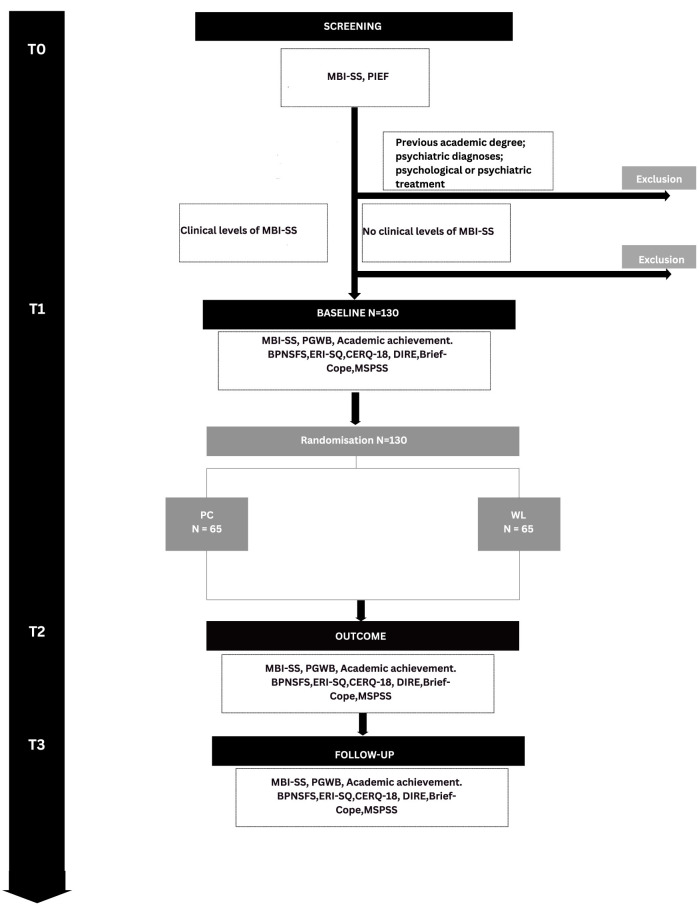
Study design and patient recruitment (N = projected numbers). MBI-SS, Maslach Burnout Inventory-Student Survey; PIEF, Personal Information and Eligibility Form (PIEF); PGWB-S, Psychological General Well-Being index-short version; BPNSFS, Basic Psychological Need Satisfaction and Frustration Scale; ERI-SQ, Effort-Reward Imbalance student questionnaire; CERQ-18, Cognitive Emotion Regulation Questionnaire-Short Version; DIRE, Difficulties in Interpersonal Emotion Regulation (DIRE); Brief-Cope, Coping Orientation to Problems Experienced; MSPSS, The Multidimensional Scale of Perceived Social Support.

The study will entail the following phases: (T0) Screening, Recruitment and Randomization; (T1) Baseline assessment (pre-intervention); (T2) Outcome Assessment (post-intervention); and (T3) Follow-up Assessment (3 months after the intervention). A summary of study design procedures is showed in Figure 1.

### Screening and recruitment

Potential participants will be invited electronically via email to students' listservs, on a weekly basis until target enrolment will be reached. In this e-mail, potential participants will receive written information on the screening aims and those who provide written consent with screening participation will fill out the PIEF and the MBI-SS. Eligible participants identified in the screening phase will be contacted by a researcher and invited to a brief assessment interview to further evaluate their eligibility based on a psychologist's rating, to give oral and written information about the clinical trial and obtain informed consent to the clinical training. Then, participants will be randomized to PC or WL groups by a colleague not involved in the study, using a computerized randomization generator. The research team will be blind to the randomized allocation.

### Outcomes data collection

Outcome variables will be assessed at T1, T2, and T3, using a Google Form. At each time, participants of the two groups will be required to fill in the eight self-report questionnaires described above (MBI-SS, PGWB-S, BPNSFS, ERI-SQ, CERQ-18, DIRE, Brief-Cope, and MSPSS). Baseline data (T1) will be collected after brief assessment interview (once informed written consent has been obtained by the participant). T2 data will be collected after the end of the last PC session. Finally, 3 months after the end of PC, participants will be contacted electronically via email and invited to fill in the Google Form to obtain T3 data.

### Intervention

The online group psychological counseling will be provided to small groups of maximum 10 participants. The intervention will consist of 6 weekly group sessions of 2 h each, based on Transactional Analysis model (Stewart and Joines, 1987; Cannavale and Castagna, 2018). Each session will be conducted by certified psychotherapists trained in Transactional Analysis and an observer co-conductor. In line with treatment goals, intervention strategies will include: (a) psychoeducation about academic-related stress, burnout, and effective strategies to contrast burnout; (b) interventions to promote self-awareness of personal reactions to academic-related stressful events, of the connection between different levels of such reactions (thoughts, emotion, bodily sensations, and behaviors), of psychological satisfaction/frustration needs, and of maladaptive habitual behaviors (e.g., learning style) which may influence academic burnout; (c) intervention to promote initial cognitive restructuring aimed at the identification of more helpful cognitive representations of academic context events (especially cognitive representations of self and others); (d) promotion of social support encouraging social sharing and adaptive social interaction in the group. See Table 1 for an overview of strategies, in-session activities, consistency with treatment goals, and the related TA concepts/techniques.

### Statistical analysis

Descriptive statistics will be reported for participant recruitment, acceptance, adherence, and dropout rates. Baseline characteristics for each group will be reported to ensure group matching on these variables. To verify the reliability of the eight questionnaires, we will compute test-retest correlations between T1 and T2, and between T1 and T3 (using Pearson's *r* coefficients).

To assess the effects of PC on the outcome variables (MBI-SS, PGWB-S, and academic achievement), mixed model regressions will be utilized, by considering Time (T1, T2, and T3) and Group (PC vs. WL) as fixed factors and subjects as the random factor. This technique will allow us to test the interaction between Time and Group. Our expectation is that PC and WL should differ at T2 and T3, but not at T1, indicating that the PC group should achieve higher scores than the WL group after, but not before, the psychological counseling intervention.

A series of path analyses will be conducted to determine whether the measures obtained from the BPNSFS, ERI-SQ, CERQ-18, DIRE, Brief-Cope, and MSPSS questionnaires mediate the differences in the outcome variables between T1 and T2, and T1 and T3. For each analysis, the exogenous variables will be the outcome measures at T1, the mediators will be the scores in the subscales of the BPNSFS, ERI-SQ, CERQ-18, DIRE, Brief-Cope, and MSPSS questionnaires, and the endogenous variables will be the outcome measures at T1. Since each questionnaire involves multiple subscales, two separate models will be computed for each mediator. For example, for the BPNSFS questionnaire, we will test whether the Autonomy Satisfaction, Competence Satisfaction, Relatedness Satisfaction, Autonomy Frustration, Competence Frustration, and Relatedness Frustration subscales will mediate the associations between the T1 and T2 (or T1 and T3) outcome measures.

We will also calculate Reliable Change Index (RCI) and Clinically Significant Change (CSC) (Jacobson and Truax, 1991) for each patient, from pre- to post- treatment and at follow-up. The RCI is computed as a ratio in which the numerator represents the difference between each subject's pretest and post-test outcome scores, while the denominator represents the standard error of the difference between the two test scores. According to Jacobson and Truax (1991), RC indices larger than 1.96 indicate that a significant change has occurred. The CSC is represented by a patient's score moving from the “dysfunctional population” range into the “functional population.” For example, pre-treatment to post-treatment of at least 2 standard deviations from the original mean (Evans et al., 1998). Finally, we will provide grouped percentages (PC vs. WL) for those individuals who reliably improved or had a clinically significant change.

The study is based on substantial evidence that psychological interventions promoting self-awareness, cognitive restructuring, and social support are effective strategies to combat burnout. This provides a strong foundation for the research. The study applies these interventions to the specific case of academic burnout, a field that is promising but still lacks strong evidence from randomized controlled trials. In the present article, we described the research protocol of the first randomized controlled trial to determine the efficacy of an online group psychological intervention specifically designed for university students with high levels of burnout. Moreover, we gave attention to several methodological issues. In line with the empirically supported psychological treatments requirements (Chambless and Hollon, 1998), we propose a between-group design with adequate statistical power, and we clearly specified sample characteristics (defined by standard measures). We also identified a large range of possible primary and secondary outcomes, and we planned the implementation of statistical analyses suitable for the understanding of different levels of effects in the data that will be collected.

Beside such strengths, the research protocol has also few weaknesses. First, we planned a waiting list design without active control conditions, such as psychological placebo and/or another treatment control groups. We opted for a waiting list design because it is widely used in standard pragmatic trials (Purgato et al., 2015) and it presents relevant ethical advantages (it allows for the provision of care to all research participants). However, due to the lack of an active control condition, our understanding of the mechanisms underlying any possible effect of the intervention will be necessarily partial (Barkauskas et al., 2005; Hart et al., 2008). This could potentially limit the internal validity of the study. Second, according to empirically supported treatments movement (Chambless and Hollon, 1998), experiments must be conducted with treatment manuals. Even if we refer to existing Transactional Analysis manuals to design the psychological intervention, due to the novelty of the field of application a dedicated manual for academic burnout is still missing (but currently in progress). This could lead to inconsistencies in the delivery of the intervention, making it harder to replicate the study and understand which specific aspects of the intervention are most effective. Third, due to the sample size and the group format only an exiguous number of psychotherapists will conduct the interventions. We will control for the effect of the provider in statistical analysis, but our sample size will not allow a complete evaluation of “therapist effects” (Crits-Christoph and Mintz, 1991). The fact that only a small number of psychotherapists will conduct the interventions could introduce therapist effects, where the outcomes are influenced by the specific therapists' skills, styles, or personalities. While the study plans to control for the effect of the provider in statistical analysis, the small sample size may not allow for a complete evaluation of these effects. Finally, while the sample size of 130 participants may be sufficient for detecting large effects, it may not be large enough to detect smaller, yet still meaningful, effects. This could limit the statistical power of the study and the ability to generalize the findings to a larger population. Future studies should overcome such limitations.

In conclusion, while the study does have a few weaknesses, such as the lack of an active control condition, the absence of a dedicated manual for academic burnout, and the limited number of psychotherapists conducting the interventions, these limitations are acknowledged and provide avenues for future research to build upon this work. Overall, the strengths of this study make it a potentially significant contribution to the field. This would draw more attention to academic organizational politics and related supporting actions.

## Author contributions

IM: Conceptualization, Funding acquisition, Methodology, Supervision, Writing – original draft. TR: Writing – original draft. FB: Supervision, Writing – review & editing. GG: Methodology, Writing – review & editing. PC: Supervision, Writing – review & editing. IP: Supervision, Writing – review & editing. RM: Supervision, Writing – review & editing. CL: Writing – review & editing. PS: Methodology, Supervision, Writing – review & editing.
